# Supplementary material for: A toxin-antidote system contributes to interspecific reproductive isolation in rice
Source: Nat Commun. 2023 Nov 18;14:7528. doi: 10.1038/s41467-023-43015-6 (PMC10657391; doi:10.1038/s41467-023-43015-6)
Supplement: Supplementary file 1 — Supplementary Information [file 41467_2023_43015_MOESM1_ESM.pdf]

A toxin-antidote system contributes to interspecific reproductive isolation in rice

You *et al.*

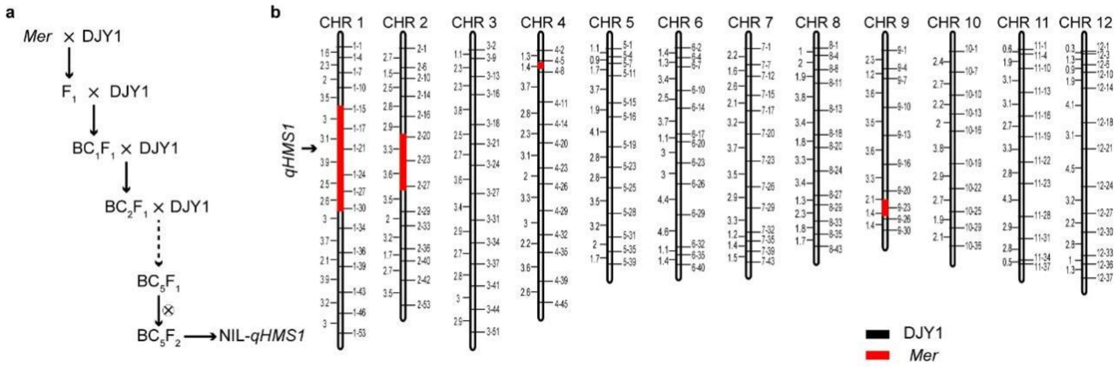

Supplementary Figure 1. Development of the near isogenic line NIL-*qHMS1*. a, Introgression of *qHMS1* from *O. meridionalis* (*Mer*) to Dianjingyou 1 (DJY1) by backcrossing to create NIL-*qHMS1*. **b**, Genomic fragments from *Mer* identified by whole-genome scanning with molecular markers. The *Mer* fragment carrying *qHMS1* associated with pollen sterility is shown (arrow). These molecular markers used for detecting *Mer* chromosomal fragment substitution are listed on the chromosome according to their relative physical position, respectively.

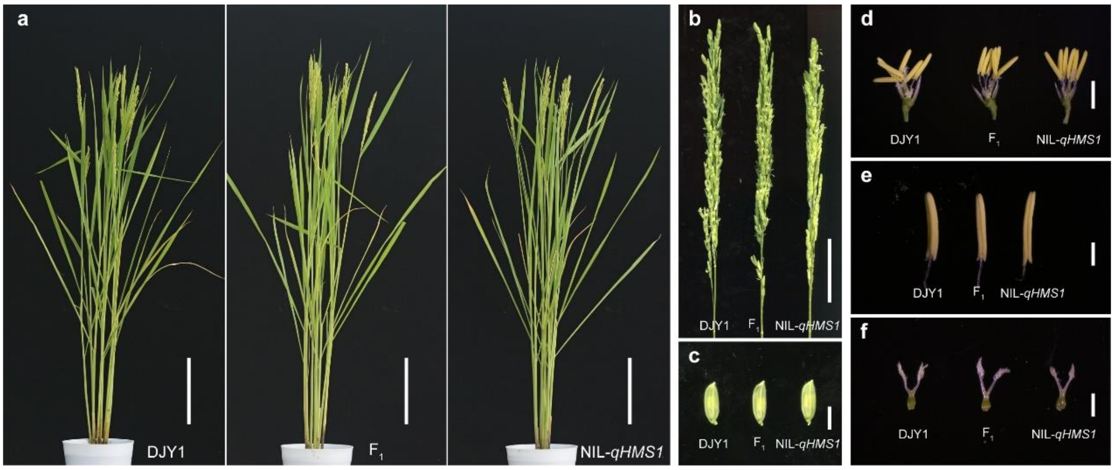

Supplementary Figure 2. NIL-*qHMS1* is morphologically similar to the recurrent parent DJY1 and their F_1. a-f, Morphology of plants at the flowering stage (**a**), panicle (**b**), spikelet (**c**), stamen (**d**), anther (**e**) and pistil (**f**) from DJY1, F_1 (DJY1 × NIL-*qHMS1*) and NIL-*qHMS1*, respectively. Scale bars, 20 cm in **a**, 5 cm in **b**, 1 cm in **c**, 5 mm in **d**, 1 mm in **e** and 2 mm in **f**. Source data are provided as a Source Data file.

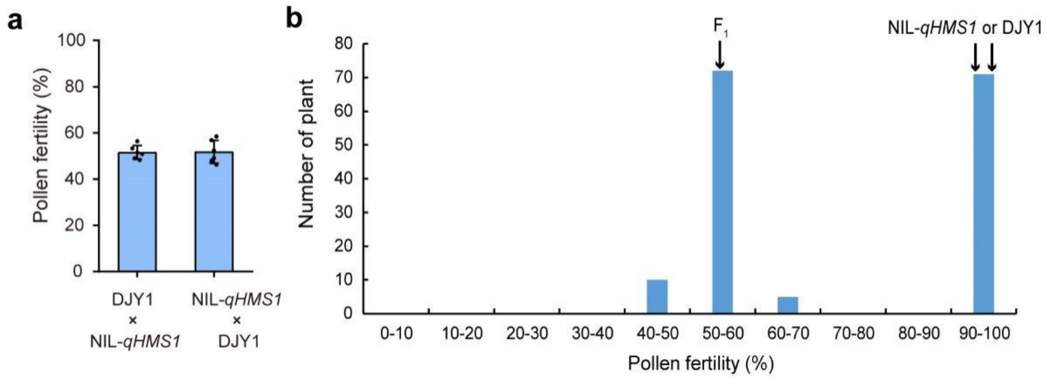

Supplementary Figure 3. Distribution of pollen fertility in the BC_5F_2 population. **a**, Pollen fertility in F_1 of reciprocal crosses between DJY1 and NIL-*qHMS1*. Data are means ± SD (*n* = 5 plants). **b**, A bimodal distribution of pollen fertility in the BC_5F_2 population of 158 plants. All the semi-sterile plants are heterozygous at *qHMS1*. Fully fertile plants are homozygous for the *Mer* allele, with very few (3 of 158) for the DJY1 allele. Source data are provided as a Source Data file.

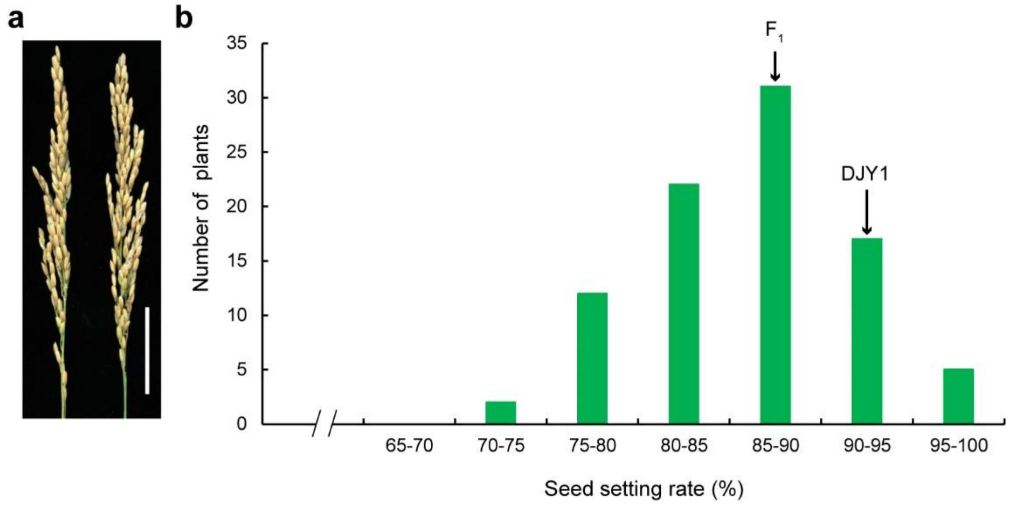

Supplementary Figure 4. Spikelet fertility of the BC_5F_2 population. a, Mature panicle of DJY1 (left) and F_1 (DJY1 × NIL-*qHMS1*) (right), showing normal seed sets. Scare bar, 5 cm. **b**, Distribution of spikelet fertility among 89 plants randomly selected from the BC_5F_2 population. Source data are provided as a Source Data file.

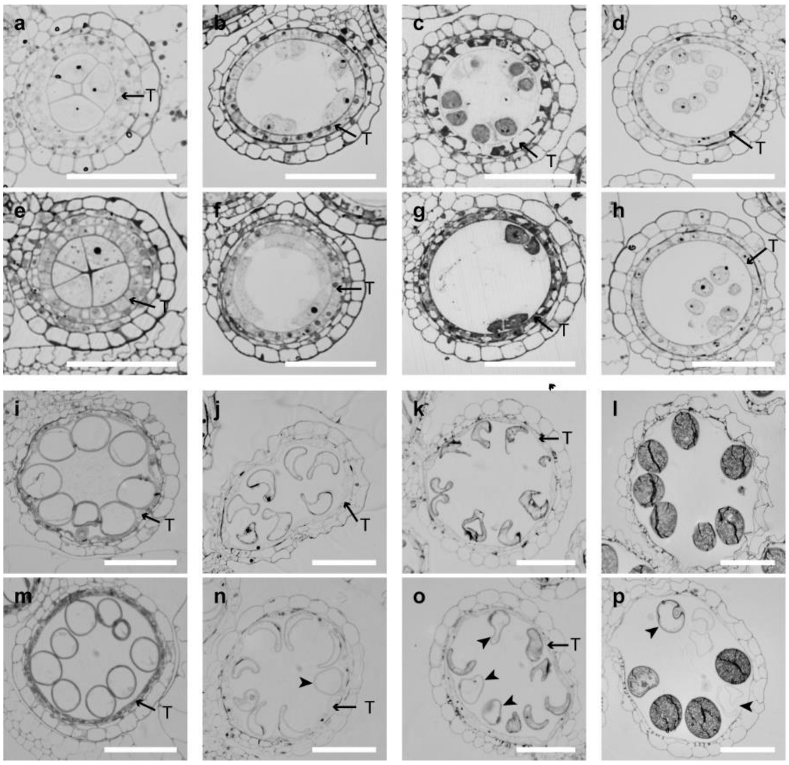

Supplementary Figure 5. Pollen abortion in anthers heterozygous at *qHMS1*. a-d and **i-l**, Transverse sections of DJY1 anthers. **e-h** and **m-p**, Transverse sections of F_1 (DJY1/NIL-*qHMS1*) anthers. Anthers were collected at meiocyte mother cell (MMC) stage (S7) (**a** and **e**), dyad stage (S8a) (**b** and **f**), tetrad stage (S8b) (**c** and **g**), early microspore stage (S9) (**d** and **h**), vacuolated microspore stage (S10) (**i** and **m**), early bicellular stage (S11a) (**j** and **n**), later bicellular stage (S11b) (**k** and **o**) and tricellular pollen stage (S12) (**l** and **p**), respectively. No obvious difference was observed between DJY1 and F_1, except aborted pollens in F_1. Arrowheads indicate aborted pollen. T, tapetum. Scare bar, 50 μm.

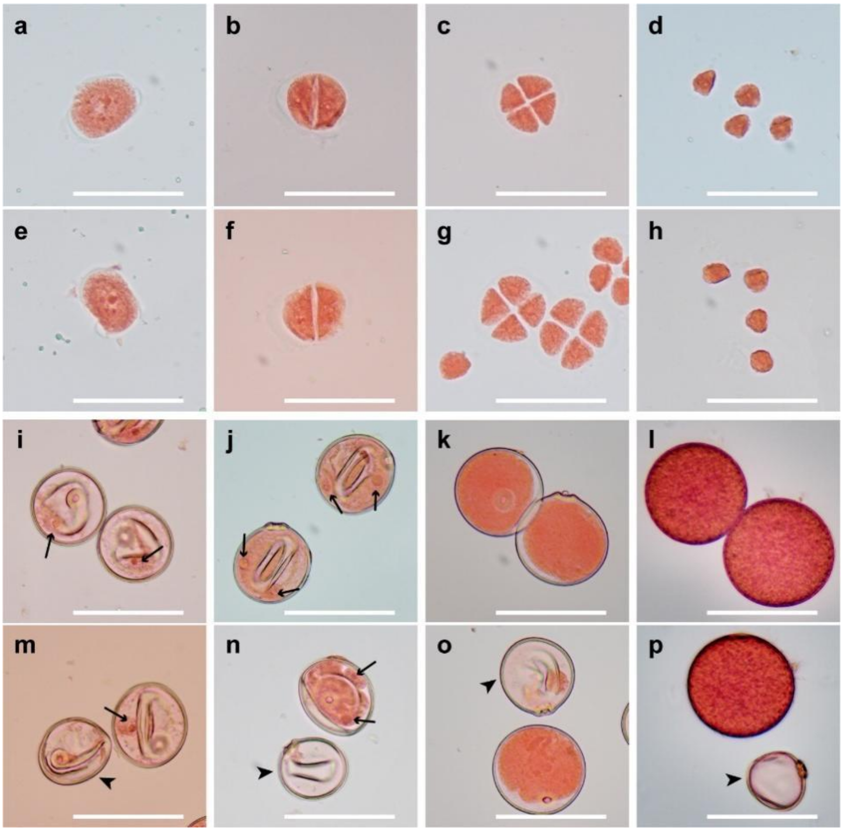

Supplementary Figure 6. Pollen development arrested at uninucleate stage in anthers heterozygous at *qHMS1*. Pollen grains of DJY1 (**a-d** and **i-l**) and F_1 (DJY1/NIL-*qHMS1*) (**e-h** and **m-p**) at different developmental stages were stained with carmine acetate. Microspores/pollens were isolated from anthers at S7 (**a** and **e**), S8a (**b** and **f**), S8b **(c** and **g**), S9 (**d** and **h**), S10 (**i** and **m**), S11a (**j** and **n**), S11b (**k** and **o**) and S12 (**l** and **p**), respectively. Partial pollens in F_1 failed to undergo first mitosis. Arrows point to nucleus. Arrowheads indicate pollen arrested at uninucleate stage. Scale bar, 40 μm.

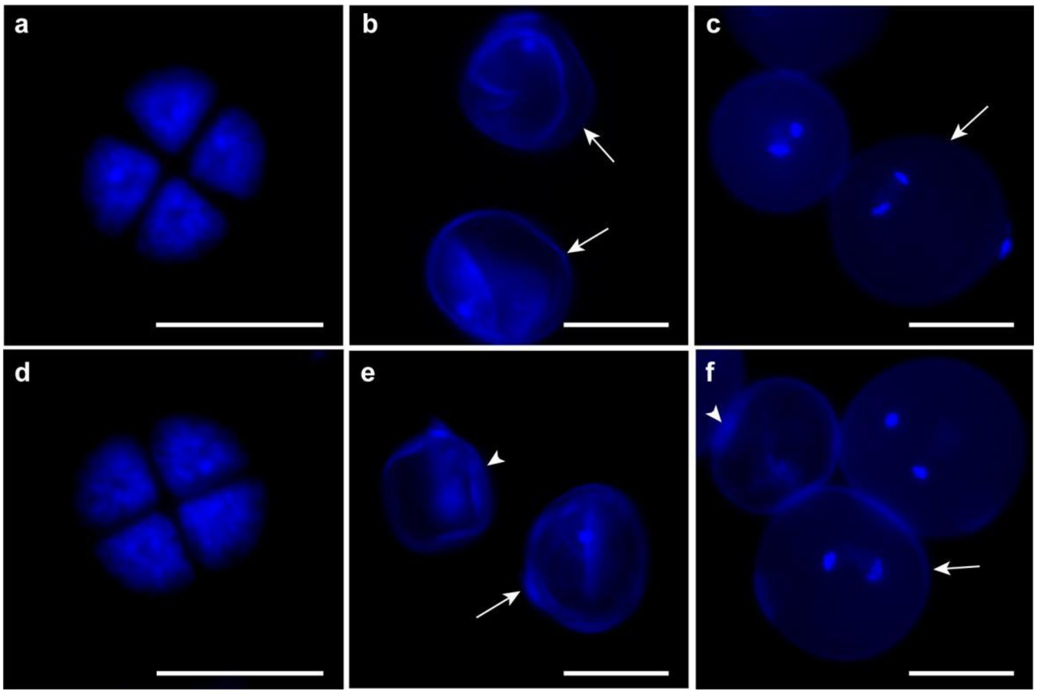

Supplementary Figure 7. DAPI staining of pollens in DJY1 and F_1 (DJY1/NIL-*qHMS1*). a-c, Pollen of DJY1. **d-f**, Pollen of F_1. Microspores/pollens were isolated from anthers at S8b **(a** and **d**), S10 (**b** and **e**) and S12 (**c** and **f**), respectively. Scale bar, 25 μm. Arrows point to normal pollen. Arrowheads point to aborted pollen without clear nuclear.

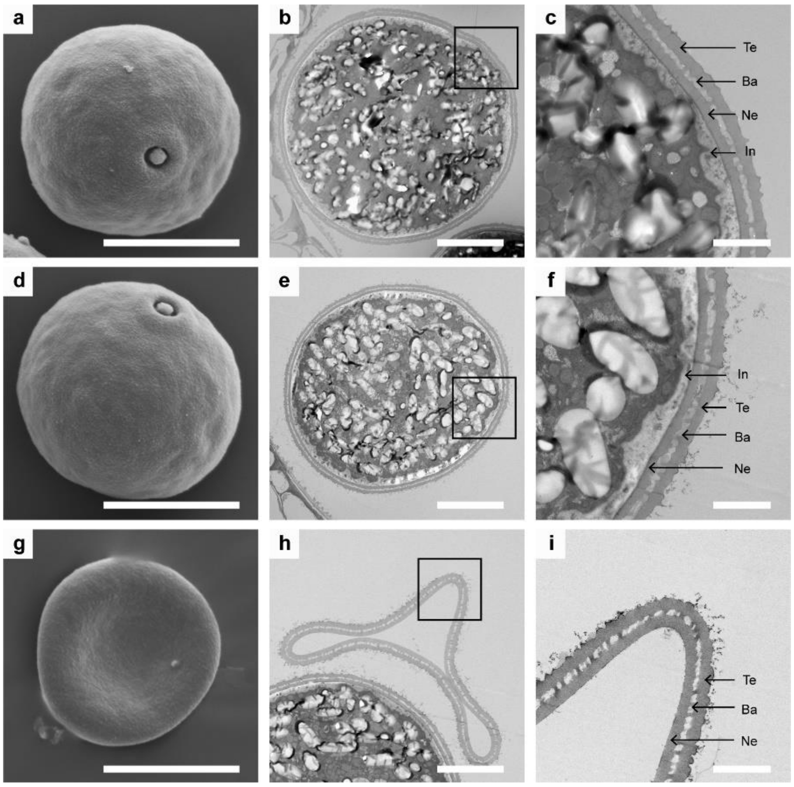

Supplementary Figure 8. Microscopic visualization of aborted pollen in F_1 (DJY1/NIL-*qHMS1*). a, Scanning electron micrograph of a fertile mature pollen from DJY1. **b**, Transmission electron micrograph of a sectioned fertile mature pollen from DJY1. **c**, Magnification of box in **b**. **d**, Scanning electron micrograph of a fertile mature pollen from F_1. **e**, Transmission electron micrograph of a sectioned fertile mature pollen from F_1. **f**, Magnification of box in **e**. **g**, Scanning electron micrograph of a sterile pollen from F_1. **h**, Transmission electron micrograph of a sectioned sterile pollen from F_1. **i**, Magnification of box in **h**. Note absence of starch granules but normal cell wall in sterile pollen. Te, tectum; Ba, bacula; Ne, nexine; In, intine. Scare bars, 20 μm in **a**, **d** and **g**; 10 μm in **b**, **e** and **h**; 2 μm in **c**, **f** and **i**.

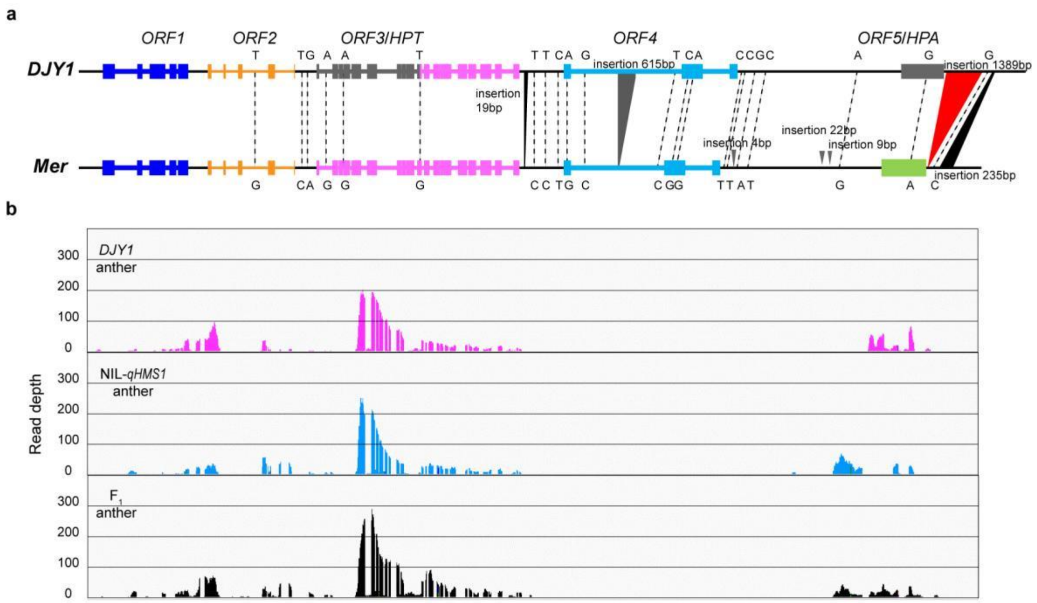

Supplementary Figure 9. Genome sequence and RNA-Seq analysis of mapping regions. a, Genome sequence alignment of the mapping region between DJY1 and *Mer*. **b**, RNA-Seq analysis of the genes at the mapping region using young anthers of DJY1, NIL-*qHMS1* and F_1. The number of mapped reads in each nucleotide is shown on the left.

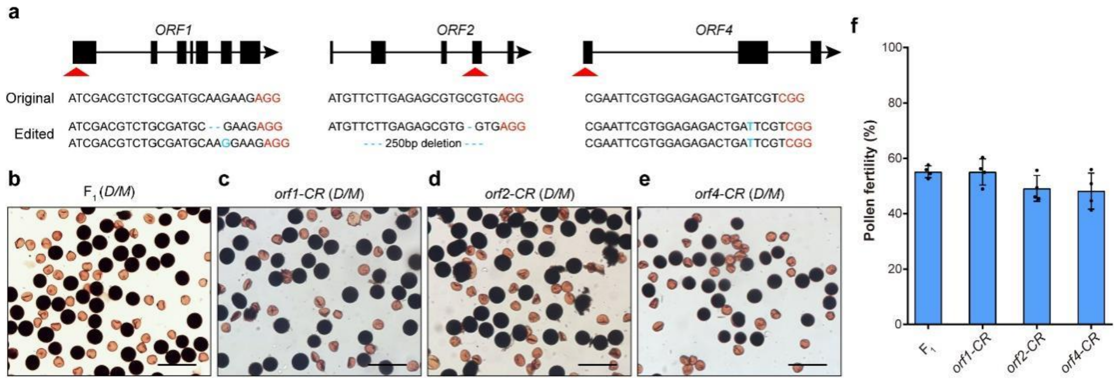

Supplementary Figure 10. *ORF1, ORF2* and *ORF4* do not contribute to hybrid male sterility. a, CRISPR-Cas9 mediated mutagenesis in *ORF1*, *ORF2* and *ORF4*. Red arrowheads indicate target site. PAM is highlighted in red and insertion or deletion mutation in blue. “Original” indicated the original sequence; “Edited” indicated the edited sequence. **b-e,** Pollen phenotype in F_1 (DJY1/NIL-*qHMS1*) (**b**), mutant line of *ORF1* (**c**), *ORF2* (**d**) and *ORF4* (**e**). *D*, DJY1 allele of *qHMS1*; *M*, *Mer* allele of *qHMS1*. Scale bar, 100 µm**. f,** Quantification of pollen fertility in **b**-**e**. Data are means ± SD (*n* = 4 independent spikelets). Scale bar, 100 µm. Source data are provided as a Source Data file.

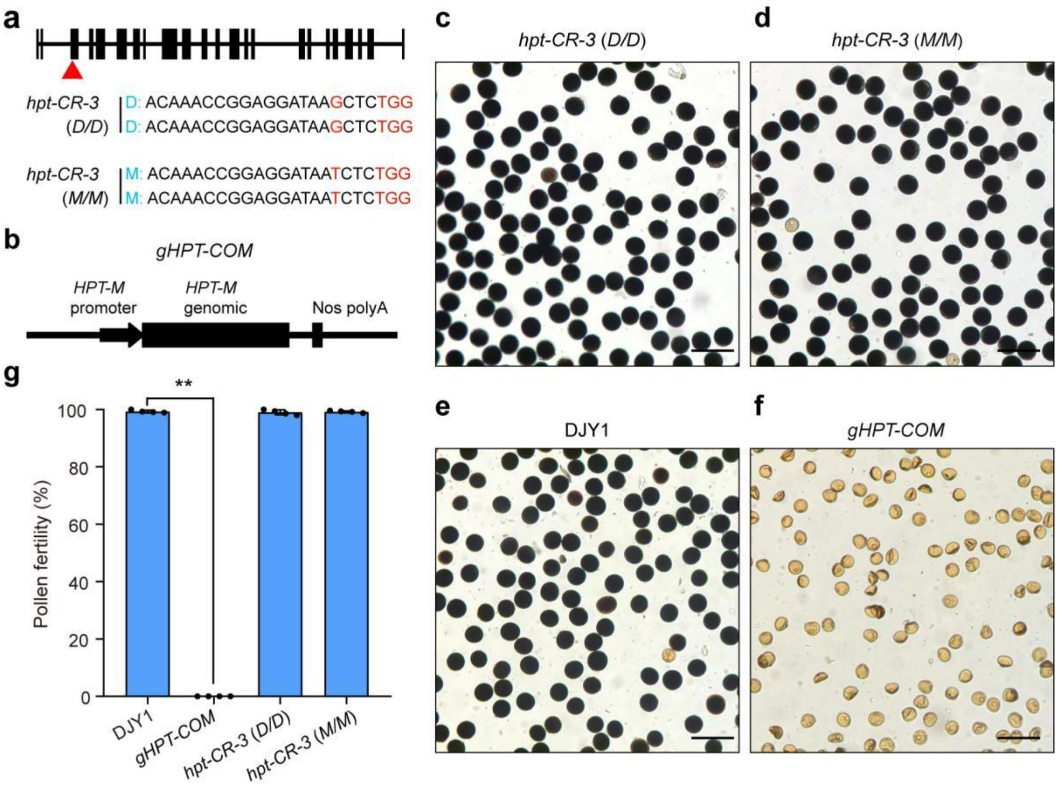

Supplementary Figure 11. Extended confirmation of HPT as a toxin. a, CRISPR-Cas9 mediated mutagenesis in DJY1 (*D/D*) and NIL-*qHMS1* (*M/M*). The PAM and base insertion in the mutant line *hpt-CR-3* are highlighted in red. **b,** Structure of an *HPT1* complementation vector (*gHPT-COM*). This vector was transformed into DJY1. **c-f,** Pollen full fertility in *hpt-CR-3* (*D/D*) (**c**), *hpt-CR-3* (*M/M*) (**d**), DJY1 (**e**) and complete sterility in *gHPT-COM* (**f**). **g,** Quantification of pollen fertility in **c**-**f**. Data are means ± SD (n = 4 independent spikelets). **P = 2.91093E-08 < 0.01 by two-tailed student's t test. *D* and *M* represent DJY1 and *Mer* origin, respectively. Scale bar, 100 μm in **c**-**f**. Source data are provided as a Source Data file.

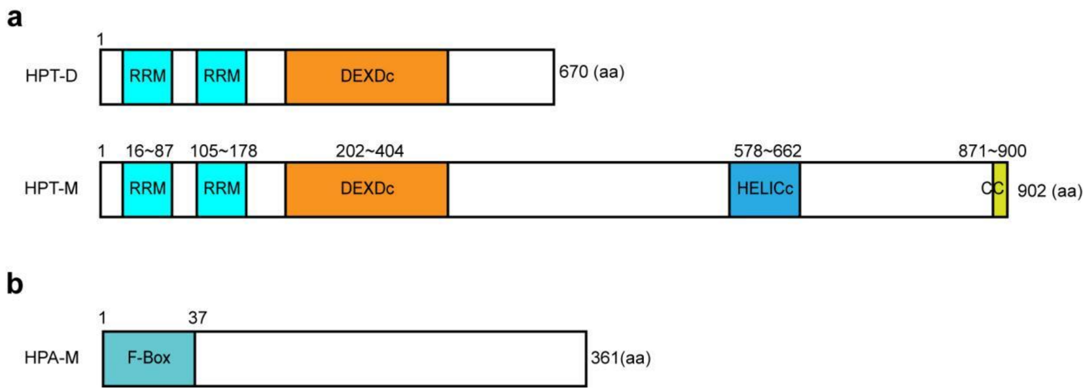

Supplementary Figure 12. Protein annotation of HPT and HPA. a, Domain annotation in HPT. HPT is truncated in DJY1 due to the early stop codon. RRM, RNA recognition motif; DEXDc, DEAD-like helicases superfamily; HELICc, helicases superfamily c-terminal domain; CC, coiled-coil. **b**, Domain annotation in HPA. D, DJY1 allele of *qHMS1*; M, *Mer* allele of *qHMS1*. Protein domains were predicted using SMART (SMART: Main page (embl-heidelberg.de)).

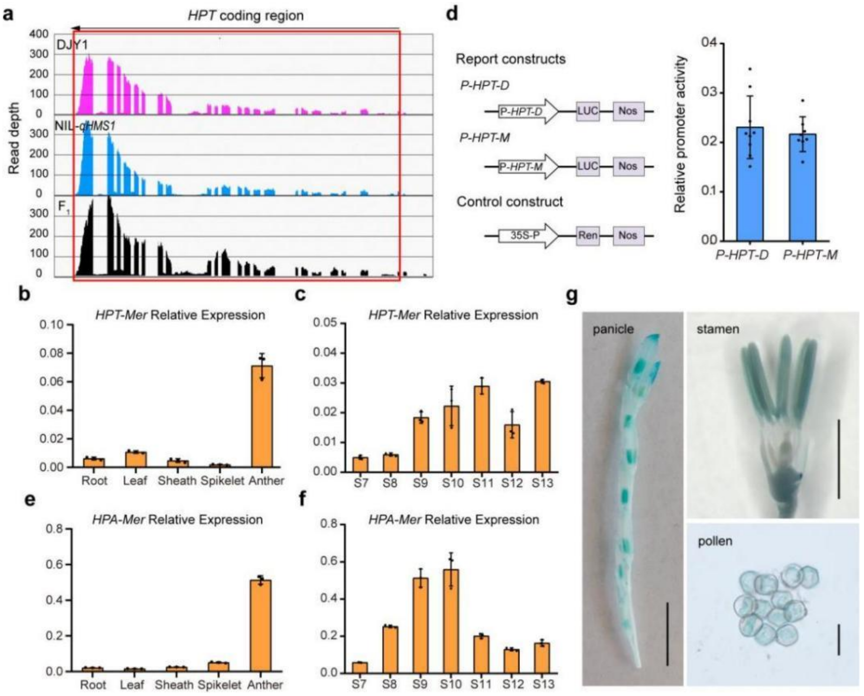

Supplementary Figure 13. Expression analysis of *HPT* and *HPA*. a, Transcript reads allocation of *HPT* reveals a similar distribution pattern between DJY1, NIL-*qHMS1* and their F_1. The development stages of the samples were at S9-S11. S9, early microspore stage (S9), S10, vacuolated microspore stage (S10), S11, bicellular stage (S11). **b, c,** *HPT* expression in different tissues collected from plants at the flowering stage (**b**) and more detailed expression analysis in pollen of different development stages (**c**). **d,** There is no difference in promoter activity of *HPT* between the DJY1 and *Mer* origins. The firefly luciferase gene (*LUC*) was used as a reporter and the Renilla luciferase gene driven by 35S promoter was used as an internal control for data normalization in protoplast transient assays. D, DJY1 origin; M, *Mer* origin. The data are means ± SD (n = 9 biologically independent samples). **e, f,** *HPA* expression in different tissues (**e**) and more detailed expression analysis in pollen of different development stages (**f**). **g,** GUS staining of transgenic panicle, stamen and pollen at early uninucleate stage. Scar bars, 1 cm for panicle, 2 mm for stamen and 40 μm for pollen. A promoter fragment of *HPA* from *Mer* was used to drive GUS expression. *UBQUITIN1* (*LOC_Os03g13170*) was used as an internal control in **b**, **c**, **e** and **f**. Data are means ± SD (n = 3 biologically replicates). Source data are provided as a Source Data file.

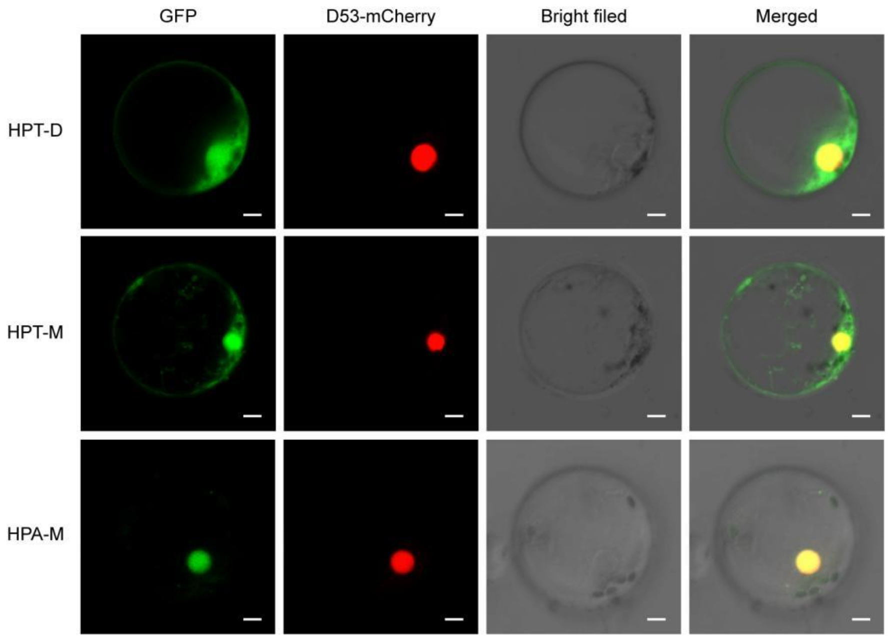

Supplementary Figure 14. Subcellular localization of HPT and HPA. *HPT* or *HPA* was fused to the *GFP* fluorescent marker and transiently expressed in rice protoplasts. HPT was localized to both nucleus and cytoplasm regardless of its DJY1 (HPT-D) or *Mer* (HPT-M) origin. HPA was localized exclusively to nucleus. D53-mCherry was co-expressed as a nuclear localization marker gene. Scale bar, 5 μm.

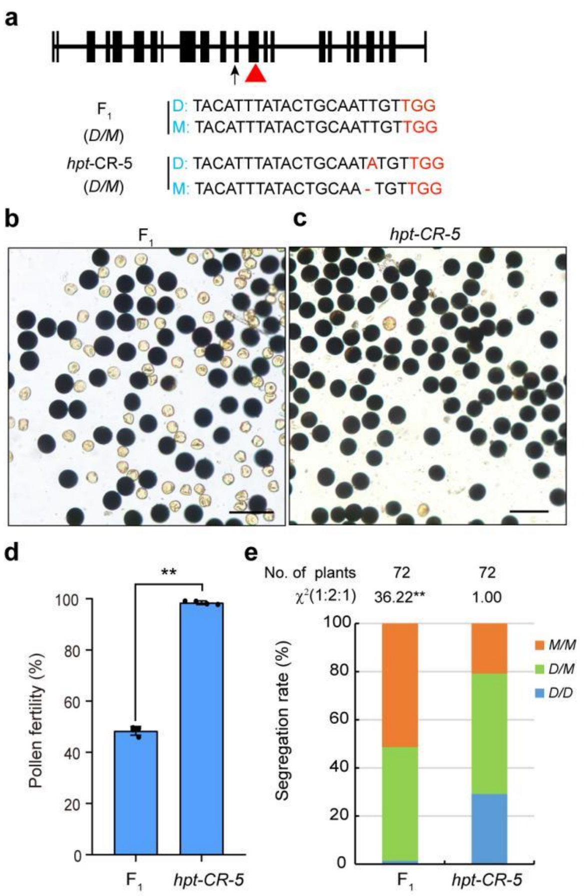

Supplementary Figure 15. The truncation event destroys toxicity of HPT. a, CRISPR-Cas9 mediated mutagenesis at a site (arrowhead) nearby the early stop SNP (arrow) in F_1 (DJY1/NIL-*qHMS1*). The PAM and base insertion/deletion in the mutant line *hpt-CR-5* are highlighted in red. **b, c,** Pollen semi-sterility in F_1 (**b**) and restored fertility in *hpt-CR-5* (**c**). **d,** Quantification of pollen fertility. Data are means ± SD (n = 4 independent spikelets). $**P = 5.36933E\text{-}06 < 0.01$ by two-tailed student's t test. **e,** Segregation ratio of progeny genotypes at *qHMS1* from *hpt-CR-5*. ($**P = 1.364E\text{-}08 < 0.01$ in χ^2 test). *D* and *M* in **a** and **e** represent DJY1 and *Mer* origin, respectively. Scale bar, 100 μm. Source data are provided as a Source Data file.

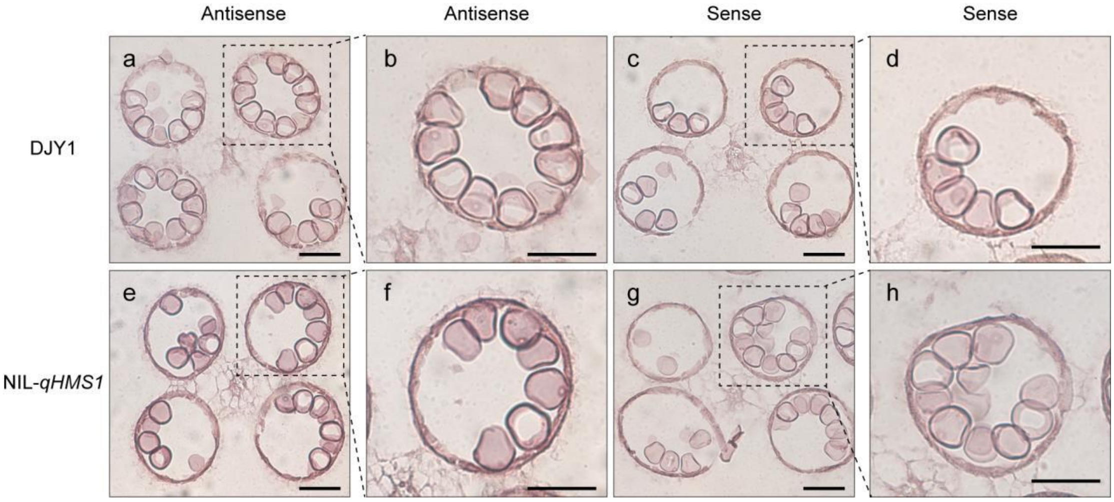

Supplementary Figure 16. RNA *in situ* hybridization analysis of *HPA*. **a**-**d**, Anther section from DJY1 was hybridized with antisense (**a**, **b**) or sense (**c**, **d**) probe. **b**, Magnification of box in **a**. **d**, Magnification of box in **c**. **e**-**h**, Anther section from NIL-*qHMS1* was hybridized with antisense (**e**, **f**) or sense (**g**, **h**) probe. **f**, Magnification of box in **e**. **h**, Magnification of box in **g**. Anthers were sampled at the uninucleate stage. Scale bar, 50 μm. Source data are provided as a Source Data file.

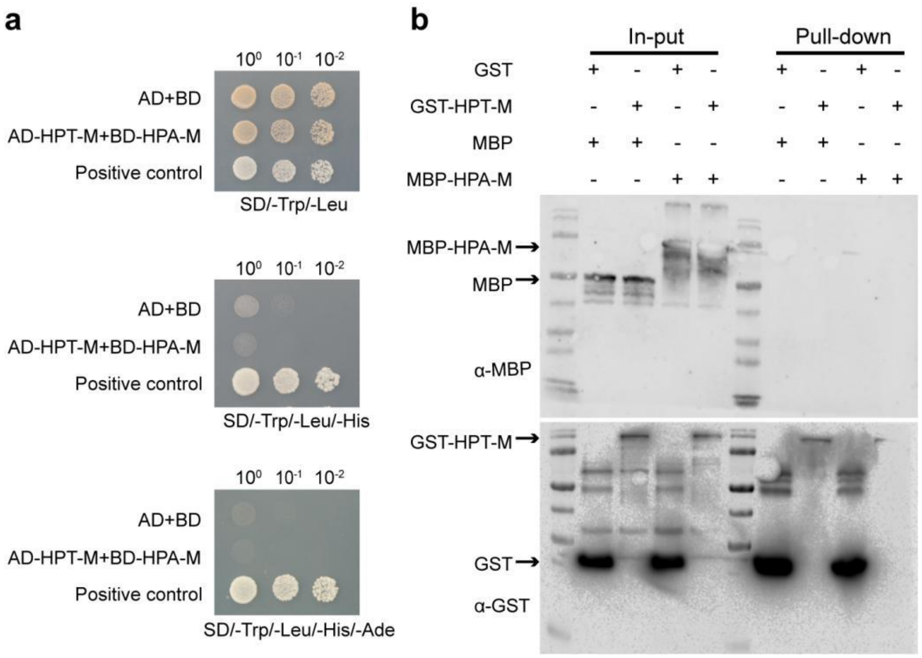

Supplementary Figure 17. The interaction of HPT and HPA. a, Validation of HPT and HPA interactions in yeast two-hybrid. **b**, Validation of HPT and HPA interactions in vitro pull-down experiments. Source data are provided as a Source Data file.

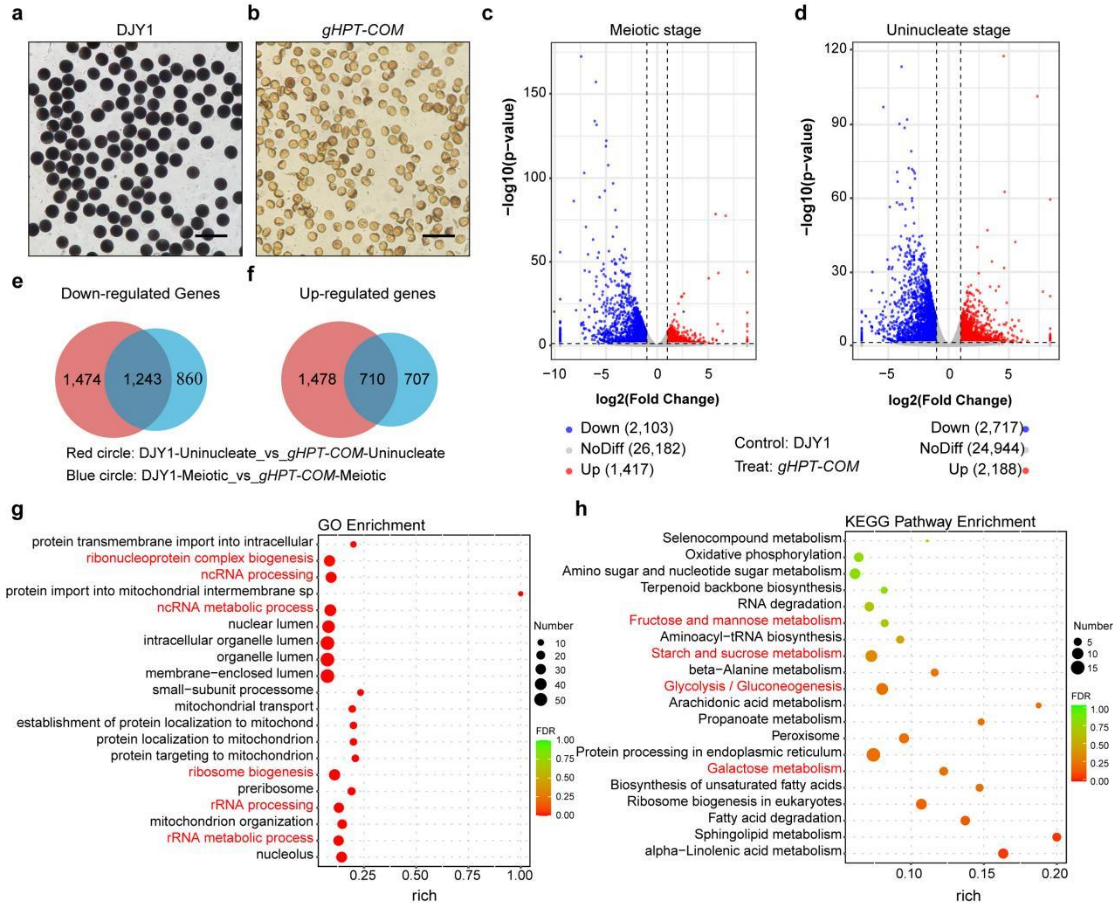

Supplementary Figure 18. Differentially expressed genes (DEGs) analysis. a, **b**, Pollen phenotype at maturity stage of DJY1 (**a**) and *gHPT-COM* transgenic plant (**b**). Scale bar, 100um. *gHPT-COM* transgenic plants were obtained by transferring *HPT* of *Mer* into DJY1 callus. Anther samples using for RNA-seq analysis were collected at meiotic stage (S8) and uninucleate stage (S9-S10). **c**, **d**, Scatter plots of differentially expressed genes (DEGs) at meiotic stage (S8-S9) (**c**) and uninucleate stage (S9-S10) (**d**) with the base 2 logarithm fold change and probability of p-value < 0.05. The expression of genes in DJY1 was treated as control. The blue dots indicate the down-regulated genes, and the red dots indicate the up-regulated genes and the grey dots indicated the genes with no difference. **e, f**, Venn of Co-down-regulated (**e**) and co-up-regulated (**f**) genes analysis between meiotic stage and at uninucleate stage. **g**, Enrichment of Gene Ontology (GO) terms of down-regulated differentially expressed genes (DEGs). **h**, Kyoto Encyclopedia of Genes and Genomes (KEGG) pathways of down-regulated DEGs.

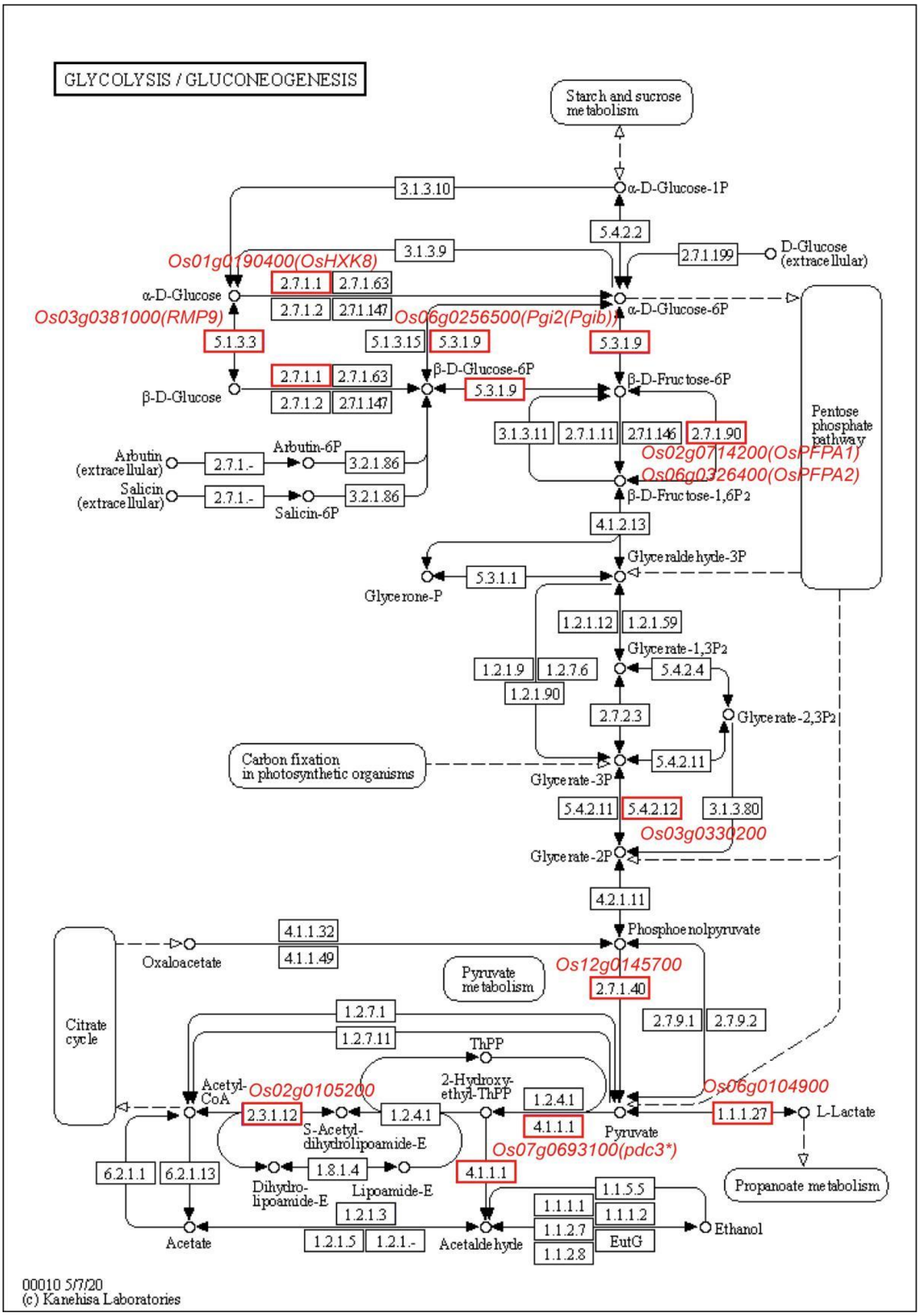

Supplementary Figure 19. Metabolic pathway of glycolysis/gluconeogenesis pathway. Metabolic pathway of glycolysis/gluconeogenesis pathway in the KEGG pathway database (http://www.genome.jp/kegg/). The down-regulated genes involved in glycolysis /gluconeogenesis pathway were marked in red.

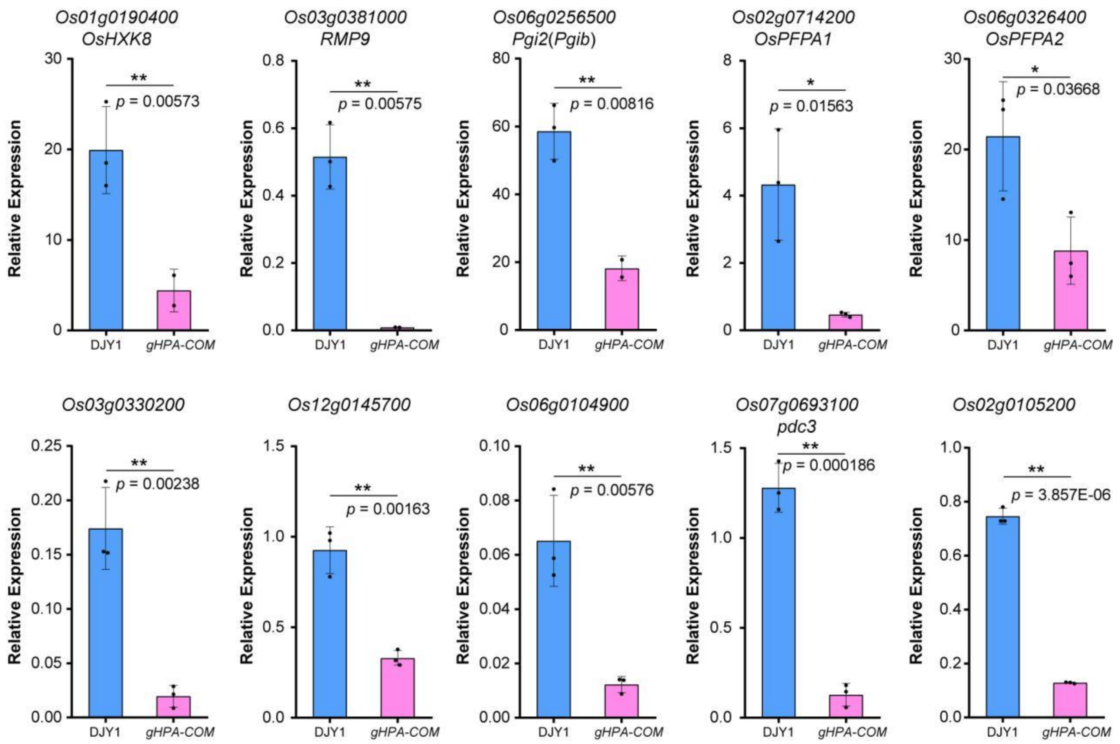

Supplementary Figure 20. Genes expression verification. qRT-PCR expression analysis of Genes involved in glycolysis/gluconeogenesis pathway. These genes were down-regulated in anther of *gHPA-COM* transgenic plants compared to DJY1. The anthers were analyzed at the meiotic stage (S8-S9) and the uninucleate stage (S9-S10). *UBQUITIN1* (*LOC_Os03g13170*) was used as an internal control. Data are means ± SD ($n = 3$ biological replicates). *$p < 0.05$ and **$p < 0.01$ in two-tailed student's t-test. Source data are provided as a Source Data file.

a

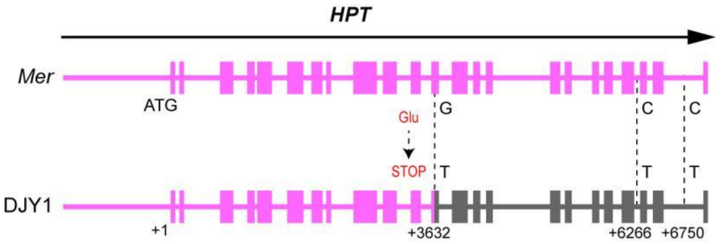

Haplotype	+3632bp	+6266bp	+6750bp	*Species (number of accessions)*
Type1	G	C	C	*O.rufipogon* (53) *O.nivara* (9) *O.officinalis* (1) *O.punctata* (1) *O.latifolia* (1) *O.meridionalis* (2) *O.longistaminata* (2) *O.sativa japonica* (7) *O.sativa indica* (16)
Type2	T	T	T	*O.rufipogon* (23) *O.sativa japonica* (21)

b

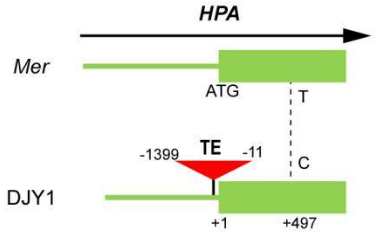

Haplotype	-11bp - 1399bp	+497bp	*Species (number of accessions)*
Type1	-TE	T	*O.meridionalis* (2)
Type2	-TE	C	*O.rufipogon* (71) *O.nivara* (9) *O.officinalis* (1) *O.punctata* (1) *O.latifolia* (1) *O.longistaminata* (2) *O.sativa japonica* (20) *O.sativa indica* (16)
Type3	+TE	C	*O.rufipogon* (5) *O.sativa japonica* (8)

Supplementary Figure 21. Haplotypes of *HPT* and *HPA* found in wild and cultivated rice of the AA genome. a, Haplotypes of *HPT*. One SNP in exon and two SNPs in intron are indicated by base position relative to the ATG start codon. **b**, Haplotypes of *HPA*. The transposon element (TE) insertion and the SNP in exon are indicated by base position relative to the ATG start codon. The tables in **a** and **b** summarize appearance of haplotypes in corresponding species of the AA genome analyzed (number of accessions in parentheses).

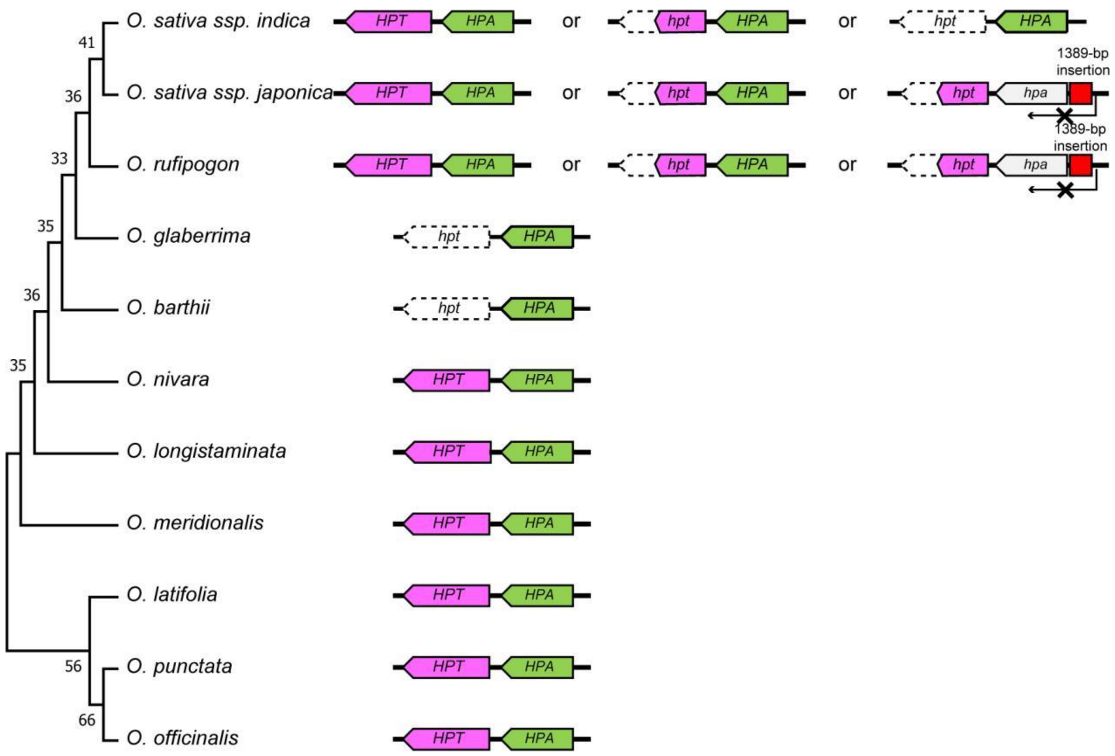

Supplementary Figure 22. *HPA* gene tree. A phylogenetic tree using the *ORF5/HPA* genome sequences from both cultivated varieties and wild rice accessions was constructed (left) and the genotype of *qHMS1* in different varieties (right).

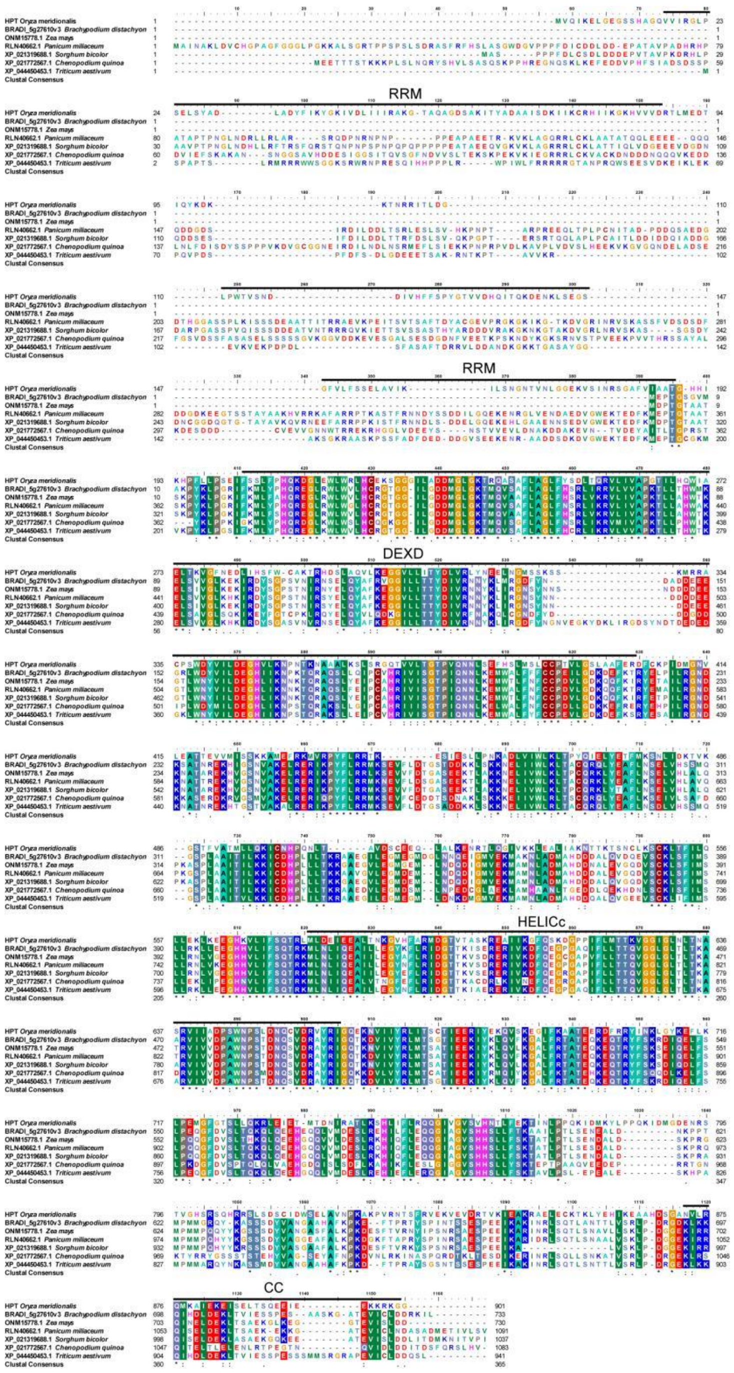

Supplementary Figure 23. HPT sequence alignment between selected species.

The black lines mark annotated domains. Identical and similar amino acids are shaded in the same color. “ - ” represents gaps in the sequences. “ * ” at bottom represents a column of identical amino acids. “ : ” at the bottom represents a column of similar amino acids. “ . ” at the bottom represents a column in which most of amino acids are identical.

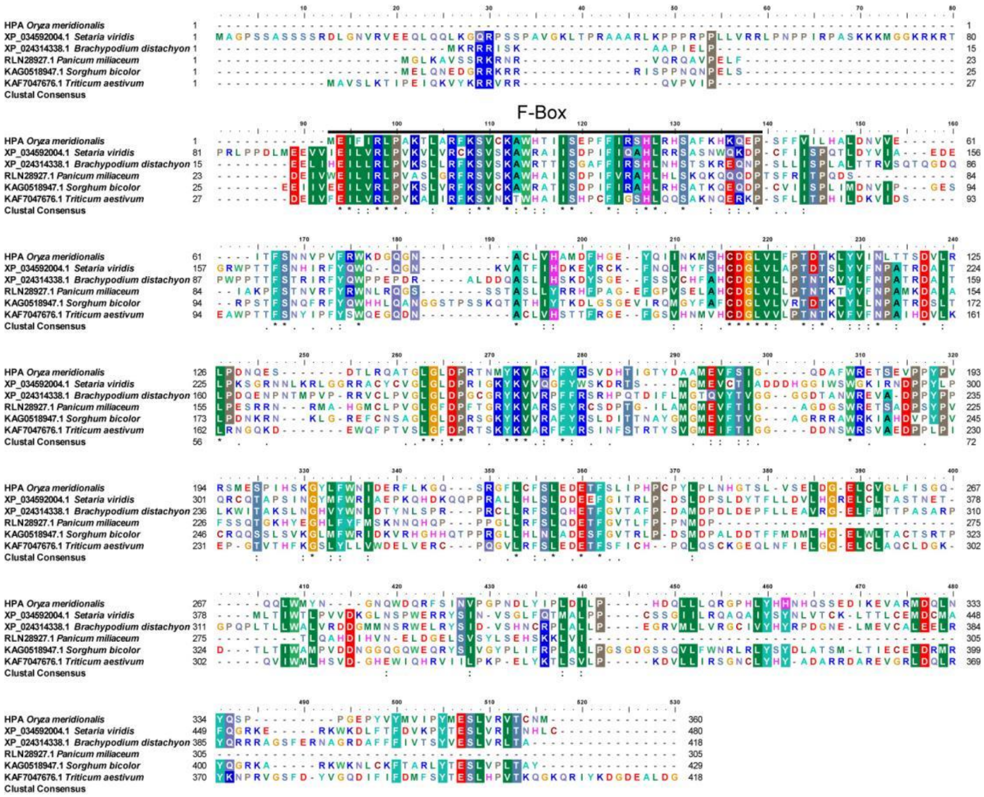

Supplementary Figure 24. HPA sequence alignment between selected species.

The black line mark the F-Box domain. Identical and similar amino acids are shaded in the same color. “ - ” represents gaps in the sequences. “ * ” at bottom represents a column of identical amino acids. “ : ” at the bottom represents a column of similar amino acids. “ . ” at the bottom represents a column in which most of amino acids are identical.

Supplementary Table 1. Predicted genes in the mapped region.

Gene name	Gene ID	Gene annotation
ORF1	*LOC_Os01g44970*	Similar to Polygalacturonase PG2
ORF2	*LOC_Os01g44980*	Peptide deformylase, chloroplast precursor
ORF3 (*HPT*)	*LOC_Os01g44990*	RNA recognition, DEAD-like helicases
ORF4	*LOC_Os01g45010*	Hypothetical protein
ORF5 (*HPA*)	*LOC_Os01g45020*	Cyclin-like F-box domain containing protein

Supplementary Table 2. Genotypic segregation ratio among T_1 progeny from selfing of *HPT* knock-out lines in F_1 (DJY1/ NIL-*qHMS1*) background.

Transgenic lines	T_1 genotype	Pollen fertility	Number of plants	Expected ratio	Expected number	χ^2 value
hpt-CR-1	*D/D*	FF	37	1	33	2.11
	D/M	FF	69	2	66	
	M/M	FF	26	1	33	
hpt-CR-2	*D/D*	FF	43	1	35.25	2.50
	D/M	FF	63	2	70.5	
	M/M	FF	35	1	35.25	
hpt-CR-3	*D/D*	FF	31	1	27.75	1.68
	D/M	FF	58	2	55.5	
	M/M	FF	22	1	27.75	
hpt-CR-5	*D/D*	FF	21	1	18	1.00
	D/M	FF	36	2	36	
	M/M	FF	15	1	18	

D, DJY1 allele; *M*, *Mer* allele; FF, fully fertile. Expected ratio means the segregation ratio according to Mendel's Law of Segregation. Expected number means the numbers from calculation based on the 1:2:1 ratio. χ^2 value, obtained by Chi-square goodness-of-fit test.

Supplementary Table 3. Genotypic segregation ratio among T_1 progeny from selfing of *HPA* complementary lines in F_1 (DJY1/ NIL-*qHMS1*) background.

Transgenic line	T_1 genotype	Number of plants	Expected ratio	Expected number	χ^2 value
gHPA-COM-1	*D/D*	32	1	32	3.75
	D/M	108	3	96	
	M/M	52	2	64	
gHPA-COM-2	*D/D*	46	1	38	3.04
	D/M	116	3	114	
	M/M	66	2	76	
gHPA-COM-3	*D/D*	23	1	22	0.55
	D/M	69	3	66	
	M/M	40	2	44	

D and *M* denote the DJY1 and *Mer* allele type respectively. *D/D* indicates DJY1 genotype plants; *M/M* indicates NIL-*qHMS1* genotype plants; *D/M* indicates heterozygous genotype plants. *gHPA-COM-1*, *gHPA-COM-2* and *gHPA-COM-3* indicates three single-cope complementary lines. Expected ratio means the segregation ratio according to Mendel's Law of Segregation. Expected number means the numbers from calculation based on the 1:2:1 ratio. The χ^2 value obtained by Chi-square goodness-of-fit test statistics.

Supplementary Table 4. Predicted genotypes among progeny of a F_1 (DJY1/NIL-*qHMS1*) carrying a single *gHPA* transgene.

♀ / ♂	Sterility<br>*D*; -	*D*;<br>*gHPA*	*M*; -	*M*;<br>*gHPA*
D; -	--	*D/D*;<br>*gHPA/-*	*D/M*;<br>*-/-*	*D/M*;<br>*gHPA/-*
D;<br>*gHPA*	--	*D/D*;<br>*gHPA/gHPA*	*D/M*;<br>*gHPA/-*	*D/M*;<br>*gHPA/gHPA*
M; -	--	*D/M*;<br>*gHPA/-*	*M/M*;<br>*-/-*	*M/M*;<br>*gHPA/-*
M;<br>*gHPA*	--	*D/M*;<br>*gHPA/gHPA*	*M/M*;<br>*gHPA/-*	*M/M*;<br>*gHPA/gHPA*

D and *M* denote the DJY1 and *Mer* allele type respectively. *D/D* indicates DJY1 genotype plants; *M/M* indicates NIL-*qHMS1* genotype plants; *D/M* indicates heterozygous genotype plants. *gHPA* indicates the complementary transgene fragment. “-” indicates null. *D/D* genotype plants were appeared in the offspring of complementary lines. Pollens of *D* genotype with transgene fragment were recused.

Supplementary Table 5. Description of these downregulated genes involved in glycolysis/gluconeogenesis pathway.

RAP locus	MSU locus	Expression	Description
Os01g0190400	*LOC_Os01g09460*	*OsHXK8*	Similar to Hexokinase
Os03g0381000	*LOC_Os03g26430*	*RMP9*	Similar to Aldose 1-epimerase-like protein
Os06g0256500	*LOC_Os01g09461*	*Pgi2(Pgib)*	Similar to Glucose-6-phosphate isomerase
Os02g0714200	*LOC_Os01g09462*	*OsPFPA1*	Similar to Pyrophosphate--fructose 6-phosphate 1- phosphotransferase alpha subunit (EC 2.7.1.90) (PFP)
Os06g0326400	*LOC_Os01g09463*	*OsPFPA2*	Phosphofructokinase domain containing protein
Os03g0330200	*LOC_Os01g09464*	-	2,3-bisphosphoglycerate-independent phosphoglycerate mutase (EC 5.4.2.1) (Phosphoglyceromutase)
Os12g0145700	*LOC_Os01g09465*	-	Pyruvate kinase family protein
Os06g0104900	*LOC_Os01g09466*	-	Similar to L-lactate dehydrogenase B (EC 1.1.1.27) (LDH-B) (Fragment)
Os07g0693100	*LOC_Os01g09467*	*pdc3**	Similar to Pyruvate decarboxylase isozyme 3 (EC 4.1.1.1) (PDC)
Os02g0105200	*LOC_Os01g09468*	-	Similar to Dihydrolipoamide S-acetyltransferase (EC 2.3.1.12)

Supplementary Table 6. Sequence similarity between HPT and its homologs in other species.

Species	Locus ID	Identity/similarity
Brachypodium distachyon	*BRADI_5g27610v3*	42.07%
Zea mays	*ONM15778.1*	46.33%
Panicum miliaceum	*RLN40662.1*	45.41%
Sorghum bicolor	*XP_021319688.1*	43%
Chenopodium quinoa	*XP_021772567.1*	41.97%
Triticum aestivum	*XP_044450453.1*	45.92%

Supplementary Table 7. Sequence similarity between HPA and its homologs in other species.

Species	Locus ID	Identity/similarity
Setaria viridis	*XP_034592004.1*	34.26%
Brachypodium distachyon	*XP_024314338.1*	34.90%
Panicum miliaceum	*RLN28927.1*	42.45%
Sorghum bicolor	*KAG0518947.1*	37.16%
Triticum aestivum	*KAF7047676.1*	42.67%
