# Supplementary material for: A toxin-antidote system contributes to interspecific reproductive isolation in rice
Source: Nat Commun. 2023 Nov 18;14:7528. doi: 10.1038/s41467-023-43015-6 (PMC10657391; doi:10.1038/s41467-023-43015-6)
Supplement: Supplementary file 3 — Description of Additional Supplementary Files [file 41467_2023_43015_MOESM3_ESM.pdf]

Description of Additional Supplementary Files

File Name: Supplementary Data 1
Description: Differentially Expressed Genes (DEGs) downregulated by 20 folds

File Name: Supplementary Data 2
Description: Accession of wild rice and Asian cultivated rice used for sequence analysis in this study

File Name: Supplementary Data 3
Description: Primers used for the scanning analysis

File Name: Supplementary Data 4
Description: Primers used in this study
